# Glycogen content and expression of CD10 and α-SMA in the microenvironment of viable human hepatic hydatid cysts

**DOI:** 10.1186/s13099-026-00797-6

**Published:** 2026-03-28

**Authors:** Enas A. El Saftawy, Marwa M. I. Ghallab, Hanaa O. Fadl, Amr M. Abdelraouf, Ahmed Naeem Eesa, Asmaa Ibrahim, Tamer Haydara, Amal M. Mahfoz, Salwa M. Morsy

**Affiliations:** 1 Department of Medical Parasitology, Faculty of Medicine, Cairo University, Cairo, Egypt; 2 Department of Medical Parasitology, Armed Forces College of Medicine, Cairo, Egypt; 3 Department of Medical Parasitology, Faculty of Medicine, Kafrelsheikh University, Kafrelsheikh, Egypt; 4 Department of Surgery, The National Hepatology and Tropical Medicine Research Institute, Cairo, Egypt; 5 Department of Pathology, Faculty of Medicine, Cairo University, Cairo, Egypt; 6 Biotechnology Research Institute, Sadat City University, Sadat, Egypt; 7 Department of Internal Medicine, Faculty of Medicine, Kafrelsheikh University, Kafrelsheikh, Egypt; 8 Egyptian Drug Authority, Cairo, Egypt; 9 Modern University for Technology and Information, Cairo, Egypt

**Keywords:** Hydatid, PAS, Glycogen, α-SMA, CD10

## Abstract

**Background:**

While hepatic cystic echinococcosis (CE) has been extensively studied in animal models, its impact on the human liver microenvironment remains unclear. Elucidating its pathogenic mechanisms in humans may reveal novel prognostic biomarkers and potential therapeutic targets. We aimed to investigate the impact of CE on the glycogen content of hepatocytes, CD10 expression in the bile canaliculi, and the profibrotic α-smooth muscle actin (α-SMA) in the adjacent portal areas.

**Methods:**

The study involved 20 cases with hepatic hydatid cysts and 20 controls. Histopathological and viability assessments were done for the surgically obtained samples. Glycogen storage was evaluated using periodic acid-Schiff (PAS) special staining. Expressions of α-SMA and CD10 were investigated using immunohistochemistry.

**Results:**

Biochemical tests and eosin staining confirmed high viability of the metacestodes, and histopathology showed distorted portal tracts. In hydatid cases, 85% (17/20) exhibited mild PAS staining of the hepatocytes, whereas control liver tissues were intensely in 65% (13/20) of the cases (*p* < 0.001). The laminated layer of the metacestode showed a strong positive PAS staining. In hydatid cases, α-SMA expression in the portal connective tissue exhibited a score of 2 in 45% (9/20) of cases, versus a score of 0 in 85% (17/20) of the control (*P* < 0.001). Increased α-SMA was also observed in the adventitial layer of the cyst. CD10 expression in pericystic liver tissue was absent in 25% (5/20) of hydatid cases, while mild to moderate canalicular expression was recorded in 65% (13/20) of cases. Nevertheless, marked expression was detected in 80% (16/20) of the controls (P < 0.001).

**Conclusion:**

As a preliminary report, hepatic hydatidosis may lead to glycogen depletion and α-SMA induction, suggesting potential therapeutic targets. CD10 may serve as a prognostic and post-treatment monitoring marker. Larger studies with multi-center design are recommended.

**Graphical abstract:**

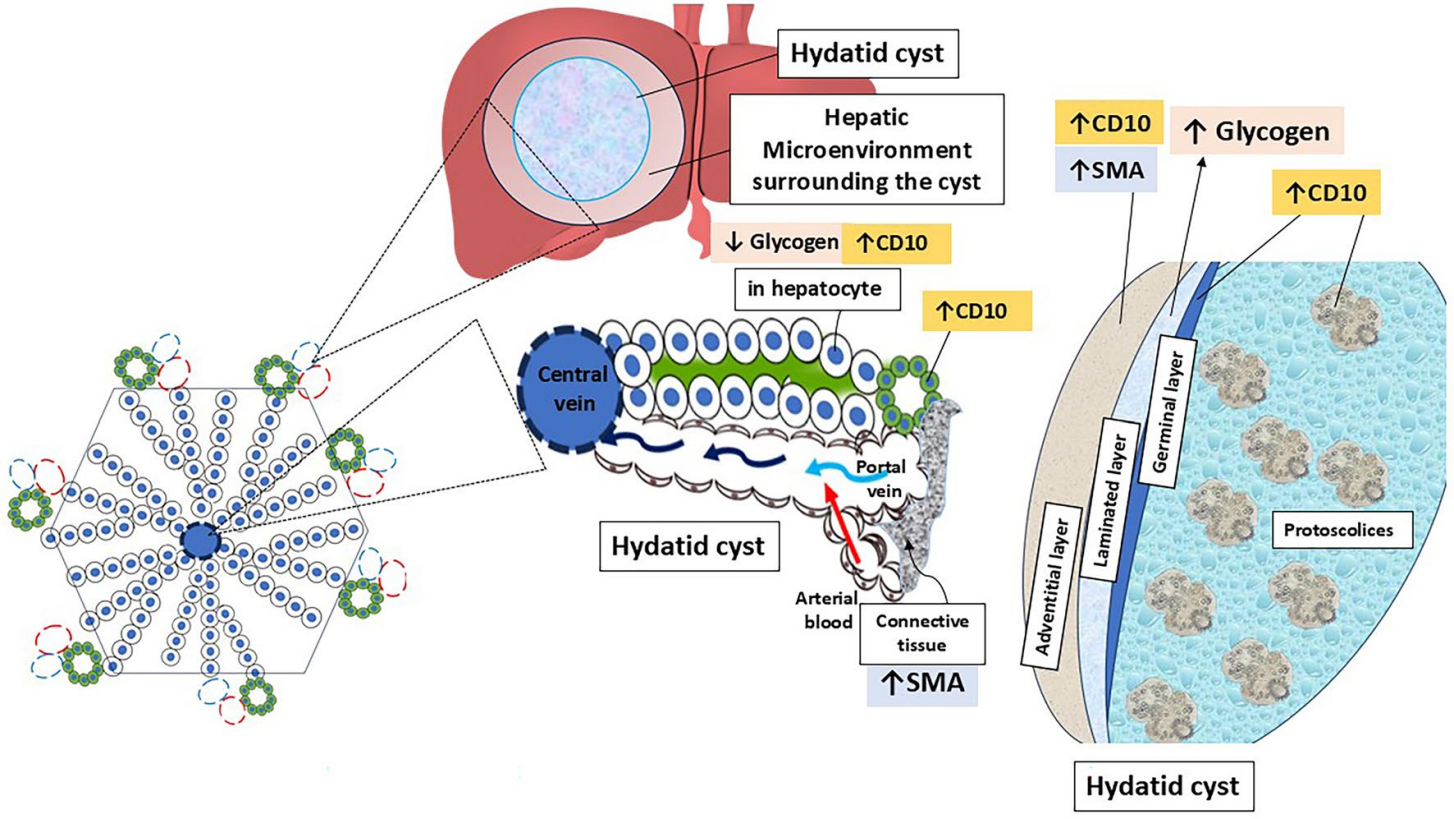

## Introduction

Hydatid disease is recognized by the World Health Organization as one of the neglected tropical diseases. It is a serious zoonotic disease that occurs in both developing and developed nations and is documented as a foremost public health problem [1, 2]. The disease is prevalent in the Middle East, Africa, Asia, Europe, and Central and South America, with more than one million people affected globally [3, 4]. Dogs are definitive hosts that harbor the adult stage of this helminth. They got infected by ingesting the viscera of the intermediate hosts that contain the larval stage. Several herbivorous and omnivorous mammals act as intermediate hosts of the larva of this parasite through ingesting contaminated legumes, or water encompassing the egg stage [5]. Humans are accidental dead-end hosts that acquire infection through a mode of infection similar to other intermediate hosts. In humans, the disease is serious as it mostly affects crucial organs such as the liver and lungs [6].

*Echinococcus* infection has been reported to induce extensive immune-inflammatory cell infiltration within the hepatic tissue, resulting in structural liver damage and subsequent fibrosis around the cyst [7, 8]. Hepatic fibrosis is a complex inflammatory condition that is triggered by persistent liver injury. If left untreated, the pathology might progress to liver cirrhosis with subsequent liver failure or a higher risk of hepatocellular carcinoma (HCC) [9, 10].

The pathology of hepatic cystic echinococcosis (CE) has been largely investigated in experimental animal studies [11, 12]. However, there is a paucity of data regarding the detrimental consequences of the disease on the liver microenvironment in human hosts. Identifying the mechanisms behind the pathogenesis of hepatic CE in humans, besides the interactions between the parasite and the adjacent liver tissue, is necessary to understand and manage echinococcosis-induced liver injury. This may aid in the discovery of novel prognostic markers and potential therapeutic targets for hydatidosis.

Hepatic stellate cells (HSCs) are well recognized as the key cells responsible for the fibrogenic process [13]**.** These cells are located in the space of Disse (perisinusoidal space between hepatocytes and sinusoidal endothelial cells), and they normally function as a vitamin A storage space [14, 15]. In response to persistent liver disease, inflammatory stimuli activate HSC and promote their differentiation to a myofibroblast phenotype. Eventually, myofibroblast forms generate alpha-smooth muscle actin (α-SMA) and excessive extracellular matrix (ECM) proteins rich in collagen Ⅰ and Ⅲ, which elicit liver scarring and fibrosis [8, 13, 16].

In the same context, the CD10 marker (common acute lymphoblastic leukaemia antigen) has been used in previous studies as an indicator of persistent hepatocellular injury and fibrosis. Additionally, it has been applied as a reliable marker for the pathological diagnosis of HCC [17, 18]. CD10 is a 100-kDa type II cell-surface metalloproteinase expressed in various normal and neoplastic tissues, including the epithelium of the liver, gastrointestinal tract, kidney, lymphoid precursor cells, and polymorphonuclear granulocytes [19–21]. In the normal human liver, CD10 is present in the membrane of bile canaliculi and interlobular bile ducts, and it is postulated to be involved in the post-secretory processing of bile [17, 22].

Although multiple immunohistochemical studies have assessed CD10 in several liver pathologies [18, 21, 22], to the best of our understanding, no prior research has precisely investigated CD10 expression in liver hydatid disease. To address this gap, the present study aims to evaluate CD10 expression in the liver microenvironment adjacent to hydatid cyst in human CE. Additionally, we investigate the impact of CE on α-SMA expression in the adjacent portal areas and on glycogen storage in the surrounding hepatocytes.

## Methodology

### Subjects

A case–control study was conducted on 20 hydatid cyst specimens from infected patients from January 2023 to December 2023. All patients were inpatients admitted to the Surgery Department, National Hepatology and Tropical Medicine Research Institute, Cairo. Clinical, radiological, and histopathological investigations diagnosed hydatid disease, and all tissue specimens were obtained after cystectomy. As confirmed by post-operative microscopic assessment, the inclusion criteria involved only highly fertile and viable hydatid cysts in Egyptian patients with no travel or treatment history. The exclusion criteria included patients who had undergone the PAIR technique or multiple rounds of Albendazole treatment, as well as cysts that tested positive for bacteria or fungi based on microbiological culturing of hydatid cyst fluid (HCF) [23]. A convenience sampling approach was used, including all eligible patients who met the inclusion criteria and were available during the study period.The Scientific Research Ethical Committee of the Faculty of Medicine, Kafrelskeikh University has approved the protocol from an ethical point of view (MKSU 50- 11—22). The committee was organized according to the Declaration of Helsinki guidelines, the International Conference of Harmonization ICH, and the United States (FWA) for the Protection of Human Subjects.

### Negative tissue control

Healthy control tissues were obtained from archived specimens at the Pathology Department, Faculty of Medicine, Cairo University, originated from liver donors for transplantation. Tissues were considered healthy based on available clinical data, absence of known liver disease, and normal histological findings. Age- and sex-matching with hydatid cases was performed retrospectively.

### Histopathological assessment of hydatid cysts

The obtained specimens were formalin-fixed and paraffin-embedded. Serial tissue sections were stained with hematoxylin and eosin for histological assessment.

### Assessment of the viability of the metacestode

HCF was evacuated into sterilized test tubes, and protoscolices, germinative shreds, and brood capsules were precipitated. Using NaCl sterile solution (0.9%), the metacestode products were washed 3 times, filtered through sterilized gauze, and left to precipitate at room temperature for 5 min. Obtained specimens were assessed for fertility and viability. Fertility was recorded as the average count of protoscoleces in 10 low-power fields (LPF). Protoscoleces were assessed for amoeboid-like motility and integral morphology by light microscopy. Using the vital stain eosin 0.1% solution dye, an exclusion test was performed, where the cyst fluid was added to an equal volume of eosin. Dead protoscoleces with lost membrane integrity absorb the stain, whereas viable ones remain unstained. The average percentage of viable protoscoleces across 10 LPF was recorded for each specimen. In addition, a urine strip test was performed to confirm the viability results, where the presence of glucose and the absence of protein indicated viable metacestode and vice versa with those cysts of low viability [24, 25]. Scoring was independently performed by two authors (HOF and EAE). Inter-examiner calibration between ANE and AMM revealed strong agreement (Cohen’s kappa = 0.87).

### Periodic acid-schiff special staining

To demonstrate intracellular glycogen in the liver tissues adjacent to the hydatid cyst wall, the PAS technique using combined Alcian blue was used in paraffin-embedded tissues. Glycogen content was evaluated semi-quantitatively according to the intensity of PAS-positive cytoplasmic staining in hepatocytes [26]. Staining intensity was graded as mild (1), moderate (2), or intense (3), corresponding to weak focal, intermediate, or strong diffuse PAS positivity, respectively. Scoring was performed by two authors (HOF and AI), then an inter-examiner calibration was conducted between ANE and EAE, with solid inter-examiner agreement (Cohen’s kappa = 0.80).

### Immunohistochemical staining of CD10 and α-SMA.

For immune-histochemical staining, epitope retrieval was done using the Tris–EDTA buffer epitope retrieval technique. The tissue sections were incubated for 10 min in a Thermo Scientific Ultra Vision hydrogen peroxide block. Thereafter, they were washed twice in a buffer. To block the non-specific background staining, Ultravision Protein Block (TA-xxx-PBQ) was applied, and tissue sections were incubated for 5 min. The primary antibodies are Recombinant Anti-CD10 antibody [Rabbit monoclonal [SP67] to CD10, (ab227640), Abcam, United States] and Anti-alpha smooth muscle Actin antibody [1A4] [Mouse monoclonal [1A4] to alpha smooth muscle Actin, (ab7817), Abcam, United States].

A secondary biotinylated goat anti-polyvalent antibody (Ultra Vision Large Volume Detection System Ant-polyvalent, HR), was then incubated with the tissue sections for 10 min at 25°C. Using a buffer, the sections were washed after each step four times. 3,3’ Diaminobenzidine DAB Chromagen (1–2 drops) was then added to 1 mL of DAB substrate and mixed by spinning. The mixture was applied to the tissue cut sections and incubated for ten min. All chemicals were obtained from United States Biological Inc. (Swampscott, MA, USA).

### Image analysis by real-time quantitative morpho-cytometry

The histopathological and morphometric assessment was done using Leica’s microscope software (Cambridge, England). The expressions of CD10 and α-SMA were assessed in 10 fields using a semi-quantitative scoring system. All values were then saved for further statistical analysis [27]. For CD10, staining intensity was scored as 0 (negative), 1 (weak), 2 (moderate), or 3 (strong), and the percentage of positive cells was scored as 0 (0%), 1 (1–25%), 2 (26–50%), 3 (51–75%), or 4 (76–100%). An immune reactive score (IRS) was calculated by multiplying the area and intensity scores [28]. For α-SMA, the percentage of positively stained HSCs was scored as 0 (no positive cells), 1 (< 10%), 2 (11–30%), 3 (31–50%), or 4 (> 50%) [29]. Immunoreactivity was scored by two authors (ANE and EAE). Inter-examiner calibration was carried out between HOF and SMM, with Cohen’s kappa of 0.85 indicating strong inter-examiner agreement. Scoring indices of CD10 and α-SMA are presented in detail in Tables 1 and 2, respectively.

**Table 1:** Scoring indices of CD10

Scoring parameter	Score	Interpretation
Staining intensity (OD)	0	Negative staining (0–0.2)
	1	Weak staining (0.21–0.5)
	2	Moderate staining (0.51–0.8)
	3	Strong staining (0.9–1)
Percentage of positive cells (area%)	0	No stained cells (0%)
	1	Few positive cells (1–25%)
	2	Moderate positive cells (26–50%)
	3	The majority of cells are positively stained (51–75%)
	4	Almost all cells are positively stained (76–100%)
IRS = area% x staining intensity	0	Negative
	1–4	Low expression
	5–8	Moderate expression
	9–12	High expression

OD: Optical density

IRS: Combined score or immune reactive score

**Table 2:** Scoring index of α-SMA

Score	Percentage of positive HSCs (area%)
0	No positive cells
1	Positively stained cells of < 10% of the mesenchymal cells
2	Positively stained cells of 11%—30% of the mesenchymal cells
3	Positively stained cells of 31%—50% of the mesenchymal cells
4	Positively stained cells of ˃ 50% of the mesenchymal cells

α-SMA**:** α-smooth muscle actin

HSCs: hepatic stellate cells

### Statistical methods

Data were coded and entered using the statistical package for the Social Sciences (SPSS) version 28 (IBM Corp., Armonk, NY, USA). Data were summarized using median and interquartile range in quantitative data and using frequency (count) and relative frequency (percentage) for categorical data. Comparisons between quantitative variables were done using the non-parametric Mann–Whitney test [30]. For comparing categorical data, the chi-square (χ2) test was performed. An exact test was used instead when the expected frequency is less than 5 [31]. Correlations between quantitative variables were done using the Spearman correlation coefficient [32]. P-values less than 0.05 were considered statistically significant. Considering post-hoc power, the limited sample size may have reduced the ability to detect small-to-moderate correlations; therefore, nonsignificant findings should be interpreted cautiously.

## Results

### Demographic data of the study population

In the current study, among 20 hydatid cases, 14 (70%) were males, and 6 (30%) were females, with a median age of 26.50. Cases of hydatid disease and controls were matched in age and sex with insignificant differences (Table 3).

**Table 3:** Demographic data of the study population

Hydatid disease (n = 20)					Control (n = 20)			
Sex	Female	Count	%		Count	%		P value
		6	30.0%		5	25.0%		0.723*
	Male	14	70.0%		15	75.0%		
Median Age		Median	1 st quartile	3rd quartile	Median	1 st quartile	3rd quartile	P value
		26.50	15.50	40.50	30.50	17.00	45.00	0.678**

Statistical tests: *Chi-square test (sex), **Non-parametric Mann–Whitney test (age)

Statistically significant at *P* < 0.05

### H and E-stained tissue cut sections

**Fig. 1:**
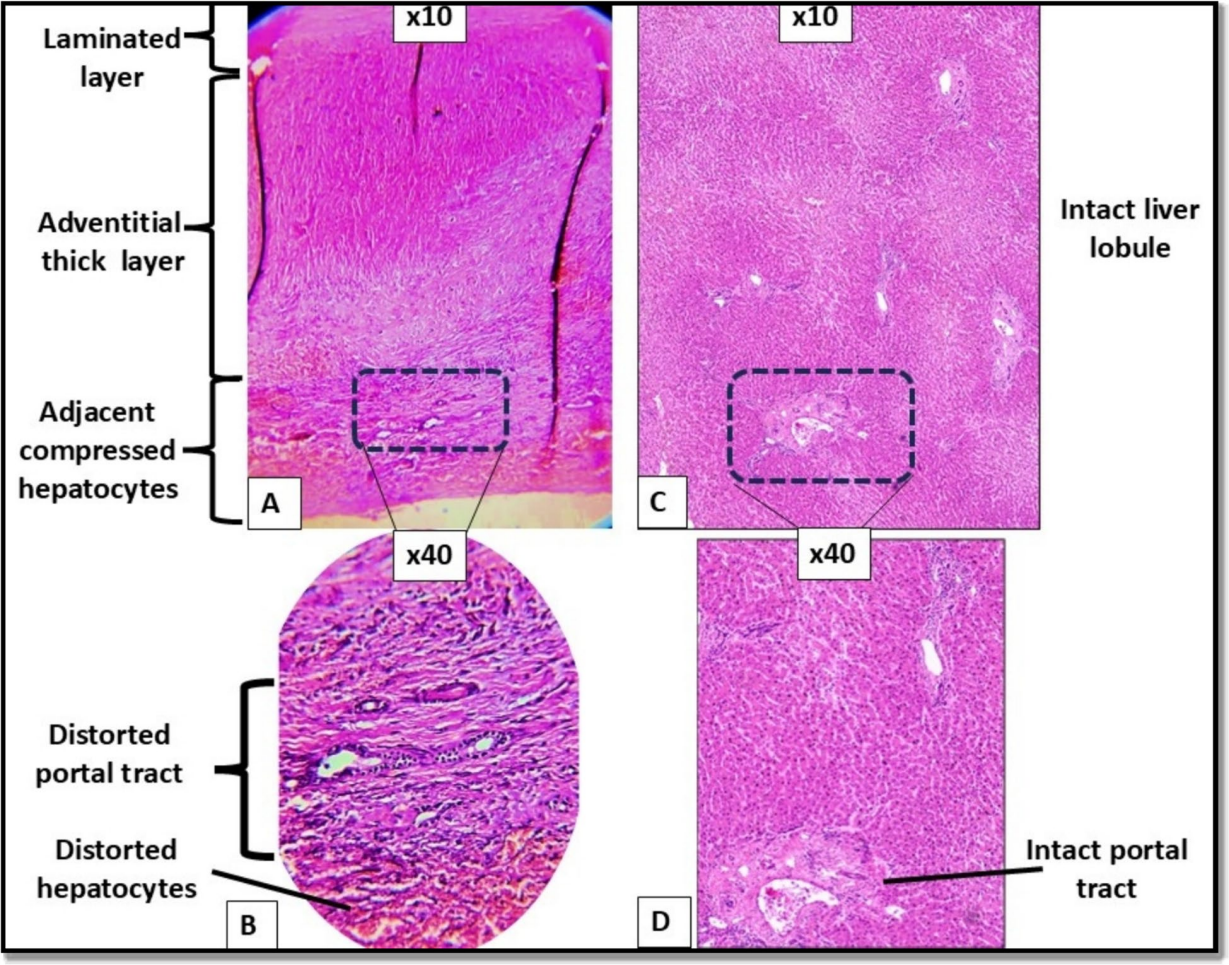
Hematoxylin and eosin-stained tissue cut sections. (**A & B**) Hepatic hydatid cyst layers. (**C&D**) The healthy control tissues

Histopathological examination of the cyst revealed an acellular laminated layer and a thick fibrous layer with infiltrating inflammatory cells (Fig. 1).

### Fertility and viability of the cyst

**Fig. 2:**
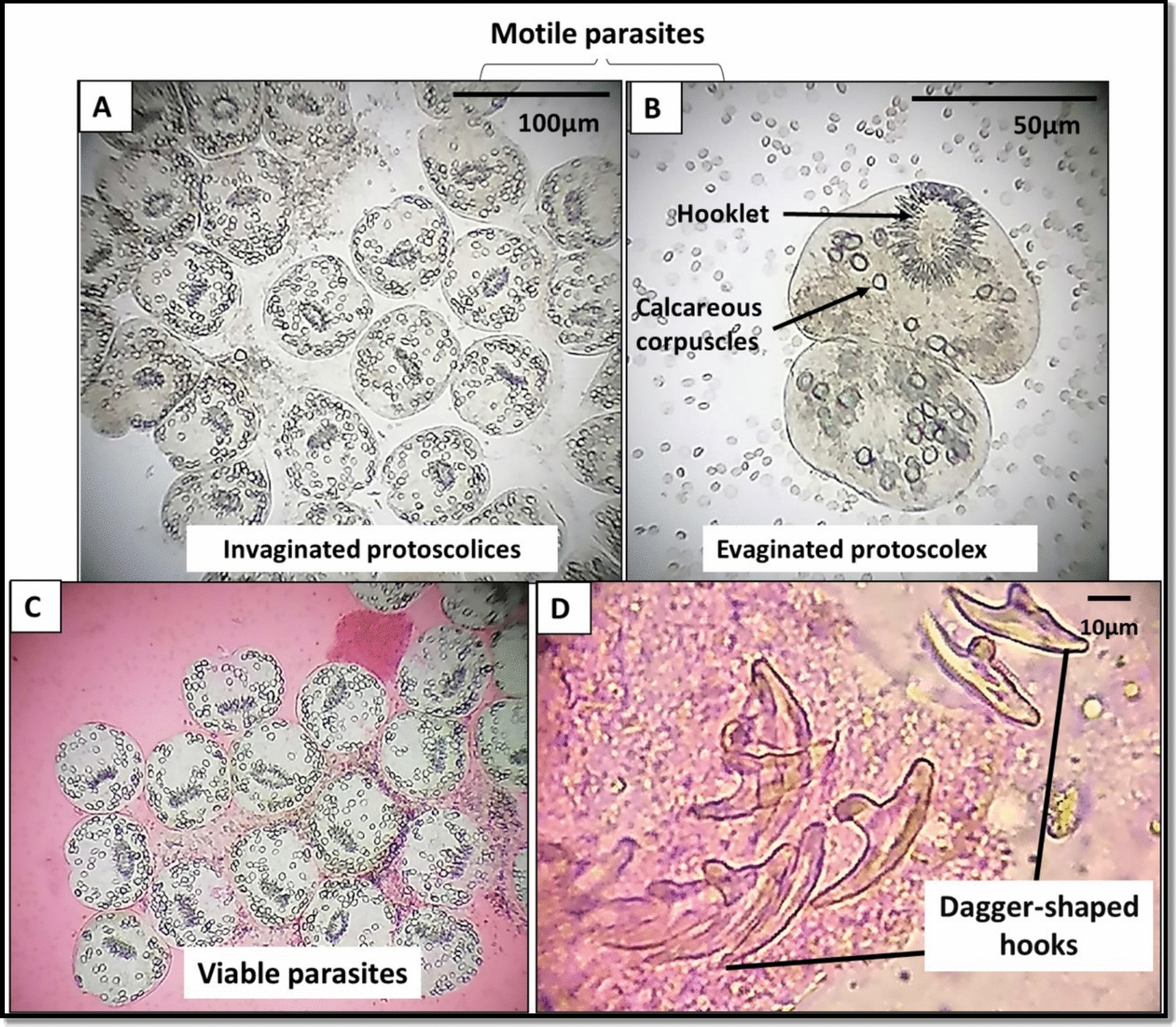
Hydatid sand obtained from the fertile hydatid cysts. (**A&B)** Motile protoscolices showing invaginated and evaginated protoscolices in a wet mount (× 100 and × 400, respectively). (**C**) Viable protoscolices excluded the eosin 0.1% solution dye (× 100). (**D**) Scattered intact dagger-shaped hooks (× 400)

The fertility results revealed a mean of 21.45 ± 5.01 protoscolices/LPF. The viability of the tested samples was determined using the eosin exclusion dye (Fig. 2), with an average of 73.60 ± 7.34% viable protoscolices/LPF. The confirmatory urine strip test revealed the presence of glucose and the absence of protein.

### PAS special stain

**Fig. 3:**
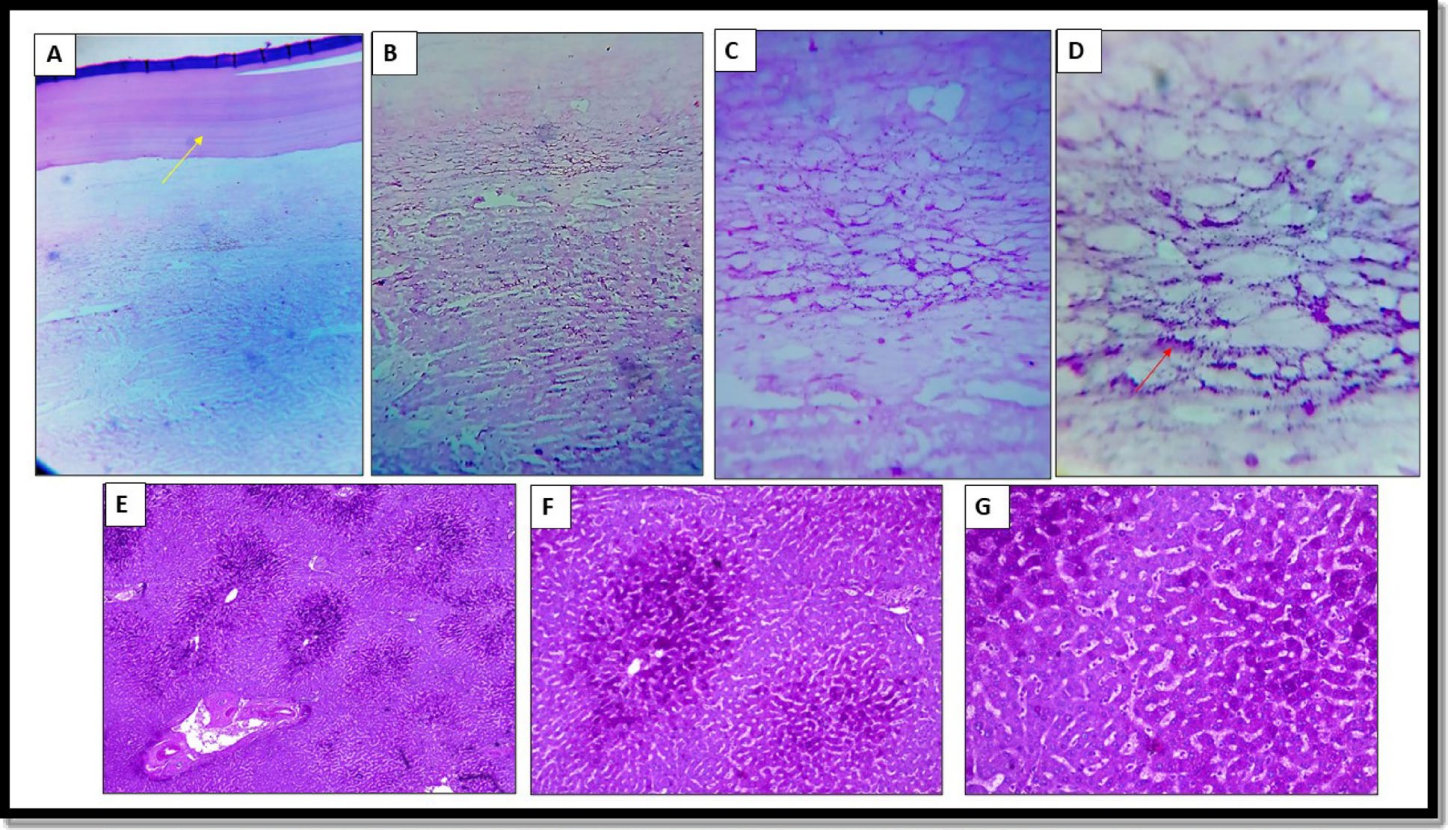
Periodic acid-Schiff (PAS) staining in the laminated layer of hydatid cyst and liver hepatocytes. (**A**) The laminated layer is intensely stained with PAS (yellow arrow) (x40). (**B, C, &D**) Scattered mild PAS-positive cells present in liver tissue adjacent to the adventitial layer (x100, x200, and x400, respectively). (**E, F, & G**) Intense glycogen accumulation was observed in the hepatocytes of the control group (x40, x100, and x200, respectively)

Among hepatic hydatidosis samples, 17 (85%) had mild PAS staining in hepatocytes, while 3 (15%) showed moderate staining intensity. The controls displayed intense hepatocyte staining in 13 (65%) and moderate staining in 7 (35%), with *P* < 0.001, Table 4. A strong PAS staining was present in the parasite’s laminated layer (Fig. 3).

**Table 4:** Assessment of the glycogen content in liver hepatocytes using PAS stain

		Hydatid disease N = 20		Control N = 20		*P* value
		Count	%	Count	%	
PAS staining scores	1	17	85.0%	0	0.0%	< 0.001
	2	3	15.0%	7	35.0%	
	3	0	0.0%	13	65.0%	

PAS: Periodic Acid–Schiff

Statistical analysis was performed using the Chi-square test

Statistically significant at *P* < 0.05

### Immunohistochemical staining of α-SMA and CD10

#### A. α- SMA expression

**Fig. 4:**
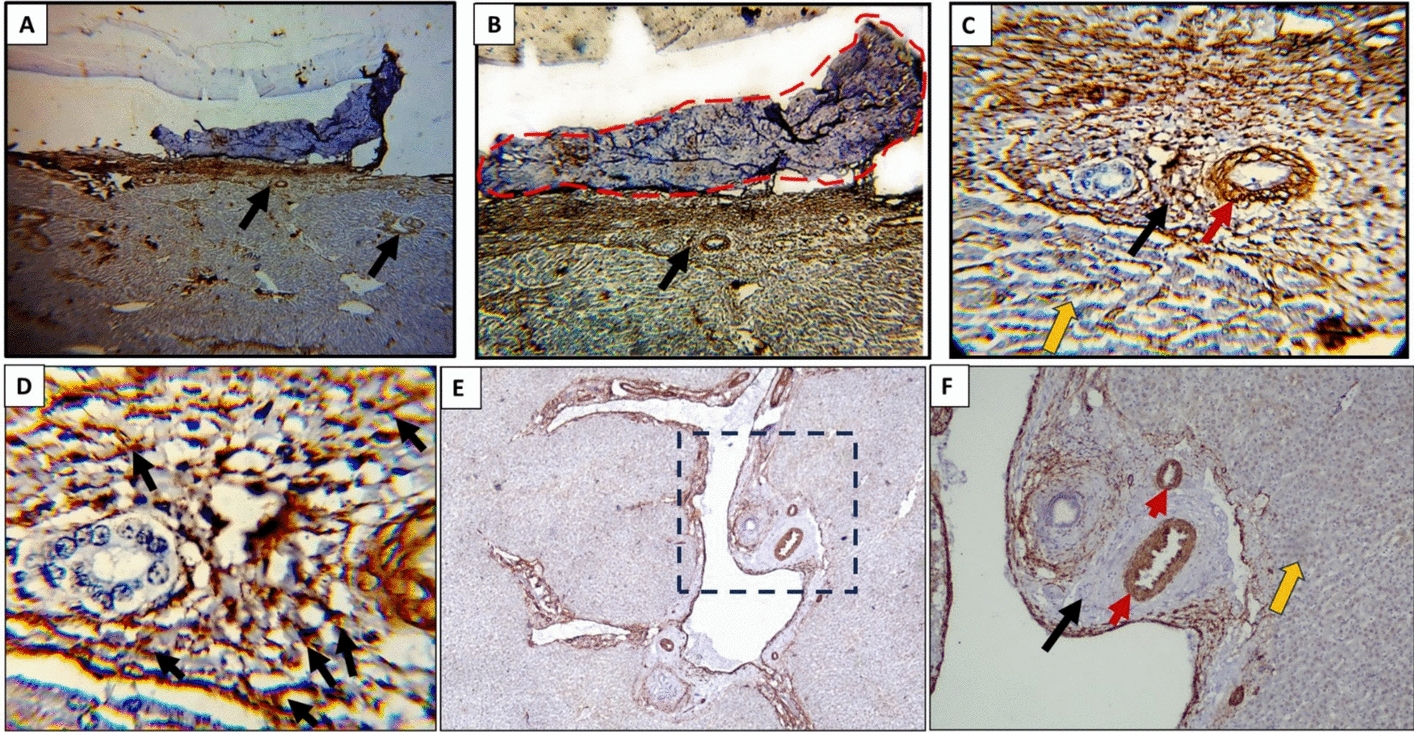
α-SMA expression in the adventitial layer and adjacent HSCs of the portal area. (**A, B**) α-SMA–positive immunoreactivity in the adventitial layer (delineated in red in B) and adjacent portal areas (black arrows); score 4 (× 40 and × 100, respectively). (**C**) Diffuse strong α-SMA staining in vascular tunica media smooth muscle cells (red arrow), portal area (black arrow), and adjacent hepatocytes (yellow arrow) near the hydatid cyst wall (× 200). (**D**) Densely stained HSCs; score 4 (black arrows) (× 400). (**E, F**) Control liver showing strong α-SMA expression restricted to vascular smooth muscle cells (red arrows), with minimal staining in surrounding portal area (black arrow) and adjacent hepatocytes (yellow arrow) (× 40 and × 100, respectively)

The α-SMA pattern in hydatid cases revealed increased expression in the adventitial layer, adjacent liver tissue, the smooth muscle of vascular structures. The expression in the normal control was limited to the smooth muscle cells of the vascular structures (Fig. 4). In hydatid cases, α-SMA expression in the connective tissue of the portal area showed a score of 2 in 45% of cases, while 85% of the controls demonstrated a score of 0 (*P* < 0.001) (Table 5).

**Table 5:** Immunohistochemical assessment of α-SMA expression in HSCs of the portal areas

α-SMA area % scores	Hydatid disease		control		*P* value
	Count n = 20	%	Count n = 20	%	
0	0	0.0%	17	85.0%	< 0.001
1	0	0.0%	3	15.0%	
2	9	45.0%	0	0.0%	
3	7	35.0%	0	0.0%	
4	4	20.0%	0	0.0%	

α-SMA**:** α-smooth muscle actin

HSCs: hepatic stellate cells

Statistical analysis was performed using the Chi-square test

Statistically significant at *P* < 0.05

#### B. CD10 expression

**Fig. 5:**
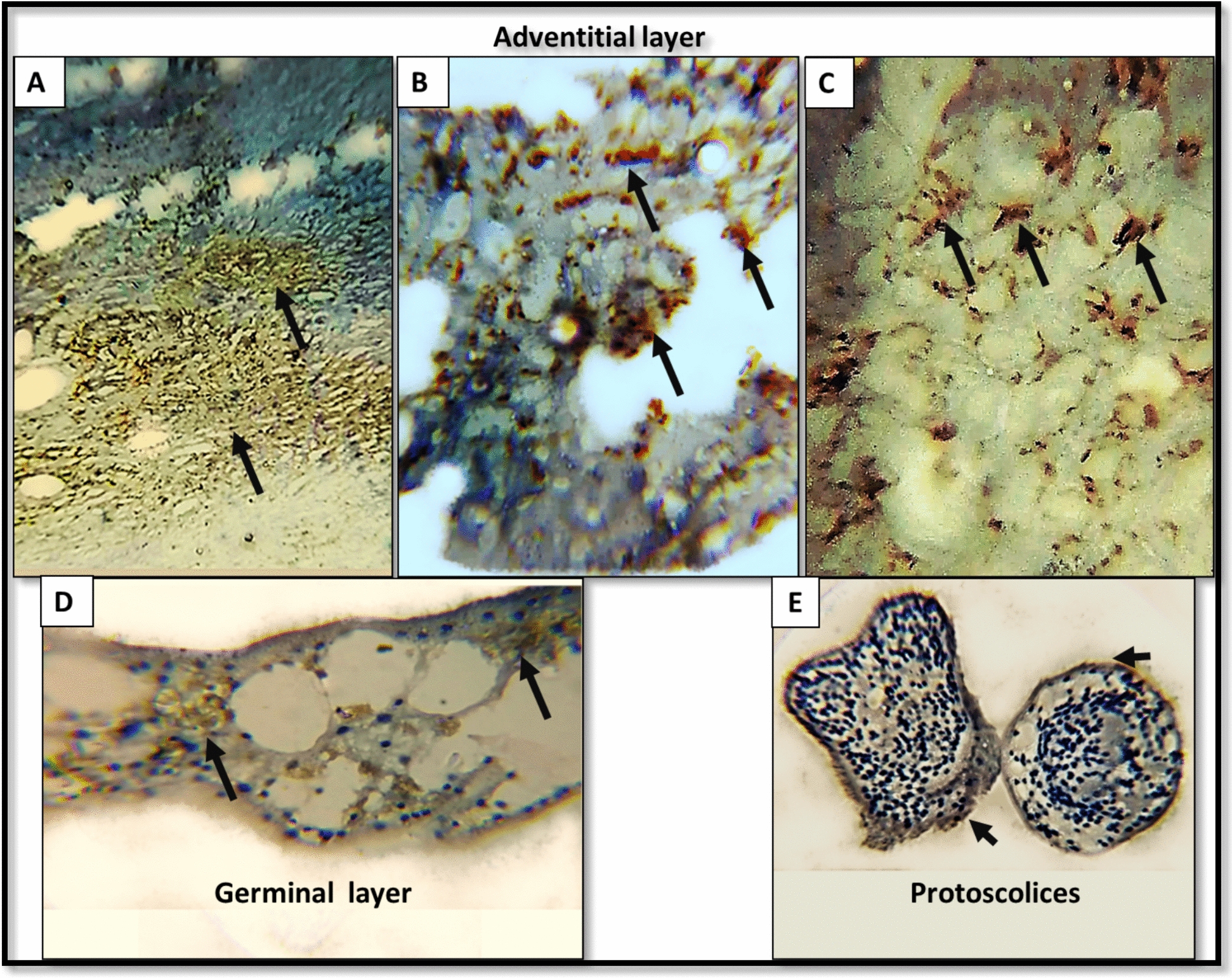
Weak to moderate expression of CD10 was exhibited in hydatid cyst structures. (**A, B, C**) Moderate cytoplasmic CD10 expression in the inflammatory cells infiltrating the adventitial layer (A: × 200 and B and C: x400x). (**D**) Weak to moderate cytoplasmic expression in the cellular structures of the germinal layer (× 400). (**E**) CD10 reactivity in the tegument of protoscolices (× 400)

**Fig. 6:**
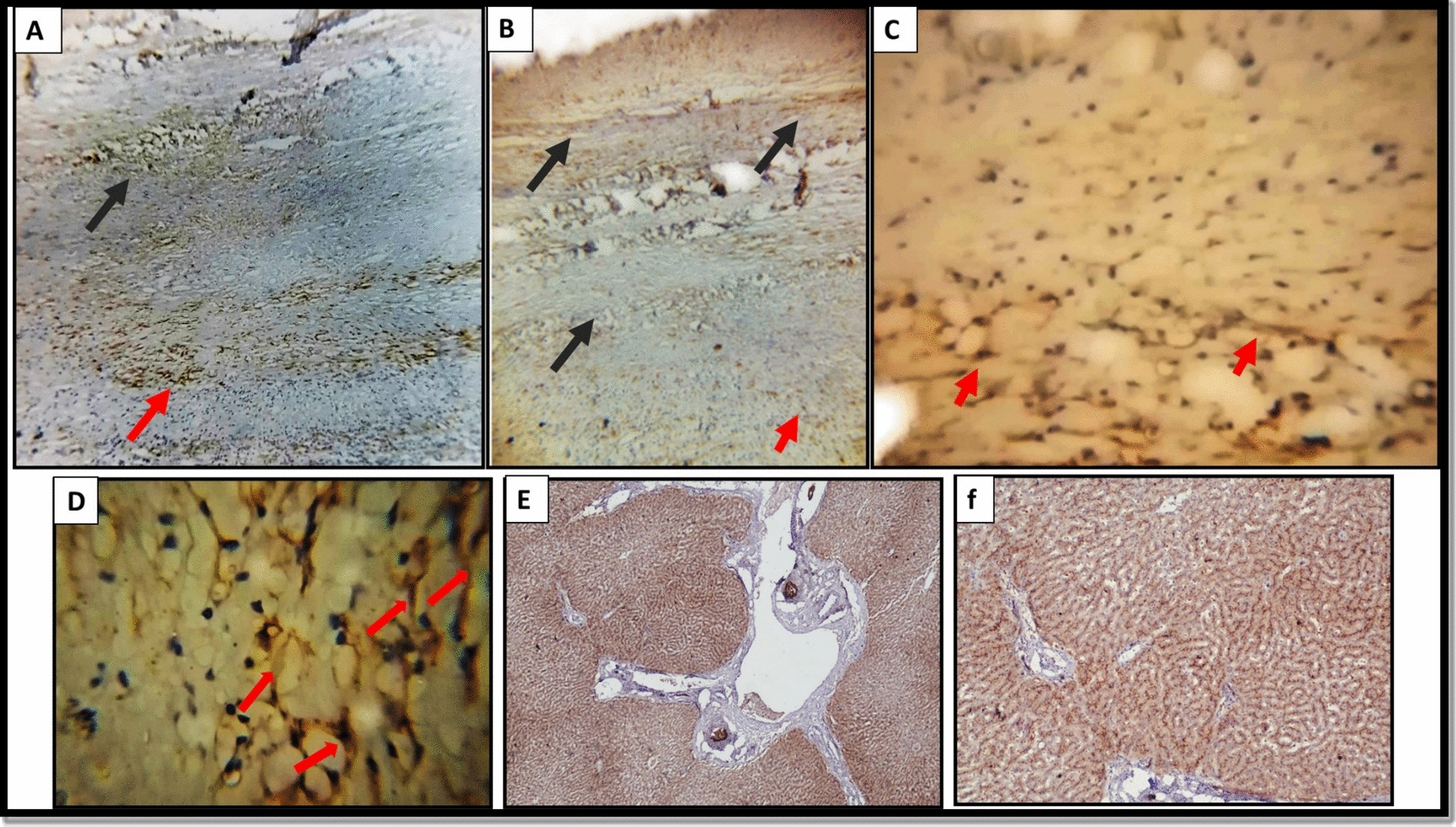
Expression of CD10 in the adventitial layer of hydatid cyst and the adjacent hepatic tissue. (**A, B**) expression of CD10 in the adventitial layer (black arrows) and the adjacent liver tissues (red arrow) (× 40 and × 100, respectively). (**C, D**) Canalicular pattern of CD10 in liver tissue adjacent to the hydatid cyst wall with low to moderate intensity (red arrow) (× 200 and × 400, respectively). (**E, F**) Control tissue with CD10 showing a diffuse strong staining canalicular pattern (× 40 and × 100, respectively)

Weak to moderate CD10 expressions were observed in hydatid tissue structures (Fig. 5). Based on IRS scoring, CD10 expression was absent in 25% of hydatid cases, while 65% demonstrated mild to moderate canalicular expression in pericystic hepatic tissue. High CD10 expression (score 12) was detected in 80% of control hepatic samples, with a statistically significant difference between the groups (P < 0.001) (Fig. 6, Table 6).

**Table 6:** Immunohistochemical assessment of CD10 expression in hepatic biliary canaliculi

		**Hydatid disease cases**		**Control**		***P*** ** value**
		**Count** (n = 20)	**%**	**Count** (n = 20)	**%**	
**Parameter**	**Scoring index**					
CD10 Average Staining Intensity score	0	5	25.0%	0	0.0%	< 0.001
	1	5	25.0%	0	0.0%	
	2	8	40.0%	5	25.0%	
	3	2	10.0%	15	75.0%	
CD10 Average area % scores	0	5	25.0%	0	0.0%	< 0.001
	2	5	25.0%	0	0.0%	
	3	7	35.0%	3	15.0%	
	4	3	15.0%	17	85.0%	
CD10 IRS scores	0	5	25.0%	0	0.0%	< 0.001
	2	4	20.0%	0	0.0%	
	3	1	5.0%	0	0.0%	
	4	1	5.0%	0	0.0%	
	6	6	30.0%	1	5.0%	
	8	1	5.0%	2	10.0%	
	9	0	0.0%	1	5.0%	
	12	2	10.0%	16	80.0%	

Statistical analysis was performed using the Chi-square test

IRS: Combined score or immune reactive score (area% x staining intensity)

Statistically significant at *P* < 0.05

### Correlations among cyst fertility, viability, α-SMA, and CD10

No significant correlation was observed among cyst fertility, viability and the area percentages of the expression of α-SMA in HSCs and CD10 in biliary canaliculi, *P* ˃ 0.05 (Table 7).

**Table 7:** Correlations among cyst fertility, viability, α-SMA, and CD10 in hydatid disease cases (n = 20)

		α-SMA	CD10
Cyst fertility/LPF	R	0.124	−0.160-
	P value	0.603	0.501
Viability %/LPF	R	0.189	−0.086-
	P value	0.424	0.717
CD10	R	0.099	
	P value	0.679	

Statistical analysis was performed using Spearman’s correlation test

*R:* Correlation Coefficient

## Discussion

El Saftawy et al. (2017) [24] demonstrated that increased glucose components in the HCF and reduced protein content are significantly present in highly viable cysts. Therefore, the current results were assumed to reflect active hydatid disease in these patients.

In the normal state, glycogen is homogeneously present throughout the hepatocytes with zonal variations attributed to metabolic enzymatic activities in the fasting or post-prandial states. Liver glycogen synthesis occurs in response to elevations in blood glucose to sustain glucose homeostasis [33]. In this study, the PAS staining showed a mild reaction in the liver tissue of hydatid- infected cases, suggesting low glycogen content in hepatocytes. Similar glycogen depletion was reported in liver pathologies such as biliary or alcoholic liver cirrhosis, attributed to the reduced activity of glucokinase [34]. Nevertheless, various effectors can mediate glycogenolysis such as glucagon, noradrenaline, and ATP. In addition, adenosine, nucleotides, and NO are glycogenolytic stimuli that act indirectly, by producing eicosanoids from non-parenchymal cells [35]. However, the precise mechanisms controlling glycogenolysis in hydatid-infected liver remain unclear.

In the current study, PAS staining in the laminated layer was strongly positive. Mucopolysaccharide is a chief constituent in the protoscolices and the laminated layer. Additionally, the literature indicates that glycogen is present in the germinal layer of the parasite [36]. A prior study showed that the addition of human insulin to hydatid larvae can enhance glucose uptake and components of insulin signaling, such as insulin receptor-like kinase and EmIR-1, in the glycogen storage cells of the metacestodes. On the other hand, insulin receptor inhibitors cause parasite killing and hamper its growth from the stem cells and insulin signaling [37]. This may reflect the possible role of the glycogen component in the survival and development of the parasite [24].

Recognizing the nutritional behavior of the parasite may serve as a therapeutic target in inoperable cysts. For instance, a combination of treatment with an important biological substance may enhance drug distribution within the cyst [38]. Chemotherapeutics such as albendazole, benzimidazole carbamates, and mebendazole restrict glucose absorption and deplete glycogen within the intracellular organelles of the parasite [39]. Loos et al. (2024) [40] showed that metformin can reduce parasite cyst weight, alter the germinal layer, and decrease the glucose available to the metacestode. This limitation of carbon and energy source hampered the parasite proliferative capacity. The study further reported that glucose depletion in the metacestode slightly upgraded the cystic uptake of 2-deoxyglucose and stimulated GLUT gene expression in the parasite. Accordingly, metformin was hypothesized as a promising treatment for hydatidosis [40].

The current study showed that the adventitial layer and adjacent liver tissue exhibited increased α-SMA expression. In addition, the study detected enhanced α-SMA expression in the vascular smooth muscle and connective tissue of the portal areas adjacent to the hydatid cyst wall. Liu GuiSheng et al. (2012) [41] demonstrated similar results for α-SMA expression in the hepatic tissues from 50 Chinese patients with hydatid cysts, where delaminated expression was observed in the outer layer of the cysts and adjacent liver tissue.

The literature showed that α-SMA is a hallmark of activated hepatic stellate cells (HSC), the key fibrogenic cells in the liver [9, 42, 43]. Nevertheless, precautions should be taken to differentiate other intrahepatic lesions that clinically simulate hydatid cysts and express α-SMA, as in chronic active hepatitis and intrahepatic biliary cystadenoma [44, 45].

Alpha-smooth muscle actin has been reported as a therapeutic target, and reducing its secretion, along with other components such as fibrotic cytokines and collagens I and III, can help lessen chronic inflammation and hepatic fibrosis [46]. Accordingly, these observations suggest that α-SMA expression levels may have prognostic value in reflecting disease severity and could be useful for monitoring therapeutic responses.

The present study demonstrated enhanced expression of CD10 in the metacestode and the surrounding adventitial layer, which, however, was low in the adjacent hepatocytes. This is the first report to assess the expression of CD10 in liver hydatidosis, providing new insight into the disease microenvironment. A prior study has shown that the CD10 canalicular pattern in the biliary canaliculi is conserved in mild fibrosis and inflammatory reactions. However, it markedly decreases with the progression of fibrosis and severe cases of lobular inflammation [47]. Further assessments of the associations between liver function tests and alterations in CD10 reactivity in liver tissue may be beneficial.

CD10 was reported to be identical to the human neutral endopeptidase, which exerts diverse physiological functions such as regulating fluid balance and inflammatory reactions [48]. During inflammatory reactions, the MAPK (Mitogen-Activated Protein Kinase) signaling pathway triggers CD10 expression that restores tissue homeostasis [49]. In contrast, Brandau and Hartl (2017) and Huapaya et al. (2023) [50, 51] reported the suppressive potential of CD10-positive neutrophils. Additionally, CD10-bearing fibroblasts were found to suppress inflammation in skin lesions [52]. CD10 was assumed to regulate inflammation by hydrolyzing various inflammatory peptides [53, 54].

Other studies also suggest that CD10, may act as a tumor suppressor in certain cancers while promoting tumor progression in others [55, 56]. Therefore, further research is recommended to investigate the potential tumorigenic role of CD10 in hepatic hydatid infection.

No apparent association was observed between parasite fertility or viability and the expression of CD10 or α-SMA; however, considering the small sample size, this may have limited the ability to detect potential associations.

Our study has limitations, including a relatively small sample size and a single-center design, which may reduce statistical power, limit the generalizability of the findings, and constrain the ability to detect small to moderate associations. Thus, the findings should be interpreted with caution. Additionally, the study lacked biochemical correlation with liver function tests and relied on paraffin-embedded tissue samples alone. Nevertheless, the results provide preliminary evidence of the impact of hydatid disease on the liver microenvironment, warranting confirmation in larger, multicenter longitudinal studies.

## Conclusion

Our study evaluated the impact of cystic echinococcosis on the liver tissue microenvironment. Compared to the control, the depleted glycogen storage in the hepatocytes adjacent to the cyst suggests a role in parasite nutrition, which may imply a potential therapeutic target in hydatid disease. α-SMA exhibited moderate increases in the hydatid cyst adventitial layer; however, compared to healthy, it showed strong expression in the adjacent hepatic portal areas. This finding proposes α-SMA as another therapeutic target in this study. This is the first study to demonstrate a preliminary characterization of CD10 expression in hepatic hydatidosis*.* Our findings reported CD10 expression in cyst structures, whereas the surrounding biliary canaliculi exhibited decreased expression compared to healthy. CD 10 might be beneficial as a prognostic and post-therapeutic monitoring marker. Further studies with larger cohorts and multi-center designs are recommended to elucidate the roles of glycogen, α-SMA, and CD10 in disease pathogenesis. Investigation of serum biomarkers and their correlations with imaging findings is also warranted.

## Data Availability

The data used and analyzed during the current study are available from the corresponding author upon reasonable request.

## References

[1] Saadi A, Antoine-Moussiaux N, Marcotty T, Thys S, Sahibi H. Using qualitative approaches to explore the challenges of integrated programmes for zoonosis control in developing countries: example of hydatidosis control in Morocco. Zoonoses Public Health. 2021;68(5):393–401. 33554481 10.1111/zph.12814

[2] Fakhri Y, Omar SS, Dadar M, Pilevar Z, Sahlabadi F, Torabbeigi M, et al. The prevalence of hydatid cyst in raw meat products: a global systematic review, meta-analysis, and meta-regression. Sci Rep. 2024;14(1):26094. 39478044 10.1038/s41598-024-77168-1 PMC11525472

[3] Borhani M, Fathi S, Lahmar S, Ahmed H, Abdulhameed MF, Fasihi Harandi M. Cystic echinococcosis in the Eastern Mediterranean region: neglected and prevailing! PLoS Negl Trop Dis. 2020;14(5):e0008114. 32379760 10.1371/journal.pntd.0008114 PMC7205190

[4] Faraj W, Abi Faraj C, Kanso M, Nassar H, Hoteit L, Farsakoury R, et al. Hydatid disease of the liver in the Middle East: a single center experience. Surg Infect (Larchmt). 2022;23(1):29–34. 34559001 10.1089/sur.2021.097

[5] Mathivathani C, Ajaykumar V, Bora CAF. Epidemiology and public health significance of hydatidosis: a review. Curr J Appl Sci Technol. 2023;42(25):19–26.

[6] Iqbal N, Hussain M, Idress R, Irfan M. Disseminated hydatid cyst of liver and lung. BMJ Case Rep. 2017. 10.1136/bcr-2017-222808. 29184009 10.1136/bcr-2017-222808 PMC5720282

[7] Zhang W, Li J, McManus DP. Concepts in immunology and diagnosis of hydatid disease. Clin Microbiol Rev. 2003;16(1):18–36. 12525423 10.1128/CMR.16.1.18-36.2003 PMC145297

[8] Labsi M, Soufli I, Amir ZC, Touil-Boukoffa C. Hepatic inflammation and liver fibrogenesis: a potential target for the treatment of cystic echinococcosis–associated hepatic injury. Acta Trop. 2022;226:106265. 34896103 10.1016/j.actatropica.2021.106265

[9] Pinzani M. Pathophysiology of liver fibrosis. Dig Dis. 2015;33(4):492–7. 10.1159/000374096. 26159264 10.1159/000374096

[10] Weiskirchen R, Tacke F. Liver fibrosis: from pathogenesis to novel therapies. Dig Dis. 2016;34(4):410–22. 10.1159/000444556. 27170396 10.1159/000444556

[11] Beigh AB, Darzi MM, Bashir S, Kashani B, Shah A, Shah SA. Gross and histopathological alterations associated with cystic echinococcosis in small ruminants. J Parasit Dis. 2017;41(4):1028–33. 29114137 10.1007/s12639-017-0929-z PMC5660029

[12] Hamad BS, Shnawa BH, Alrawi RA. Immunohistochemical and histopathological characterization of immune changes in the host-tissue reaction site of murine cystic echinococcosis. Adv Anim Vet Sci. 2022;10(11):2367–75.

[13] Zhang CY, Yuan WG, He P, Feng F, Liu H, Pi HF, et al. Liver fibrosis and hepatic stellate cells: etiology, pathological hallmarks and therapeutic targets. World J Gastroenterol. 2016;22(48):10512–22. 28082803 10.3748/wjg.v22.i48.10512 PMC5192262

[14] Friedman SL. Hepatic stellate cells: protean, multifunctional, and enigmatic cells of the liver. Physiol Rev. 2008;88(1):125–7. 18195085 10.1152/physrev.00013.2007 PMC2888531

[15] Vatankhah A, Halász J, Piurko V, Barbai T, Rásó E, Tímár J. Characterization of the inflammatory cell infiltrate and expression of costimulatory molecules in chronic *Echinococcus granulosus* infection of the human liver. BMC Infect Dis. 2015;15(1):1–12. 26578348 10.1186/s12879-015-1252-x PMC4647452

[16] Atmaca HT, Gazyagci AN, Terzi OS, Sumer T. Role of stellate cells in hepatic echinococcosis in cattle. J Parasit Dis. 2019;43(4):576–82. 10.1007/s12639-019-01129-z. 31749527 10.1007/s12639-019-01129-z PMC6841830

[17] Nakamura T. Simultaneous changes in expression of bile canalicular CD10 and sinusoidal CD105 (Endoglin) in chronic hepatitis and liver cirrhosis. In: Hepatocellular Carcinoma-Future Outlook. IntechOpen; 2013.

[18] Agarwal A, Handa U, Kundu R, Sachdev A, Kochhar S. Hepatocyte paraffin-1, CD10, and CD34 immunostaining as a diagnostic aid in cytologic diagnosis of hepatic cancer. J Cancer Res Ther. 2022;18(Suppl 2):S434-8. 36510999 10.4103/jcrt.JCRT_467_20

[19] McIntosh GG, Lodge AJ, Watson P, St-Georges A, Ross P, Gown AM, et al. NCL-CD10-270: a new monoclonal antibody recognizing CD10 in paraffin-embedded tissue. Am J Pathol. 1999;154(1):77–82. 9916921 10.1016/S0002-9440(10)65253-4 PMC1853426

[20] Chu PG, Ishizawa S, Wu E, Weiss LM. Hepatocyte antigen as a marker of hepatocellular carcinoma: an immunohistochemical comparison to carcinoembryonic antigen, CD10, and alpha-fetoprotein. Am J Surg Pathol. 2002;26(8):978–88. 12170084 10.1097/00000478-200208000-00002

[21] Rocken C, Licht J, Roessner A, Carl-McGrath S. Canalicular immunostaining of aminopeptidase N (CD13) as a diagnostic marker for hepatocellular carcinoma. J Clin Pathol. 2005;58(10):1069–75. 16189153 10.1136/jcp.2005.026328 PMC1770740

[22] Byrne JA, Meara NJ, Rayner AC, Thompson RJ, Knisely AS. Lack of hepatocellular CD10 along bile canaliculi is physiologic in early childhood and persistent in Alagille syndrome. Lab Invest. 2007;87(11):1138–48. 17876294 10.1038/labinvest.3700677

[23] El Saftawy EA, Abd-Elaal AA, Badawi MA, Abdelraouf AM, Shoeib EY, Mohsen A, et al. Viability of hepatic hydatid cysts in relation to their parasitological, microbiological and radiological features in patients treated by different protocols. J Egypt Soc Parasitol. 2021;51(2):313–22.

[24] El Saftawy EA, Abd El-Aal AA, Badawi M, Attia SS, Abdelraouf A, Shoeib EY, et al. Research note. One minute, intraoperative assessment of the viability of hydatid cysts using a simple reagent strip test. *Helminthologia*. 2017;54(2):157–64.

[25] El Saftawy EE, Abdelraouf A, Elsalam MA, Zakareya P, Fouad A, Albadawi EA, et al. Autoimmunity in human CE: Correlative with the fertility status of the CE cyst. Helminthologia. 2022;59(1):1–17. 35601761 10.2478/helm-2022-0011 PMC9075880

[26] Reddy M, Kumar NG, Manyam R, Swetha P, Supriya AN, Bharath TS. Comparison of glycogen positive cells in oral smears with random blood sugar levels of type 2 diabetes patients. Ann Med Health Sci Res. 2018;8(1):1–5.

[27] Abdelhamid GA, Abdelaal AA, Shalaby MA, Fahmy MEA, Badawi MA, Afife AA, et al. Type-1 diabetes mellitus down-regulated local cerebral glial fibrillary acidic protein expression in experimental toxoplasmosis. J Parasit Dis. 2023;47(2):319–28. 37193484 10.1007/s12639-023-01573-y PMC10182235

[28] Mohd Danil N, Siriwardena BSMS, Goh YC, Tilakaratne WM. Expression of interleukin 1-alpha, interleukin 6 and CD 10 in predicting recurrence of ameloblastoma. Discover Oncol. 2025;16(1):1390. 10.1007/s12672-025-03235-2 PMC12283532 40696210

[29] Elzamly S, Agina HA, Elbalshy AEL, Abuhashim M, Saad E, Abd Elmageed ZY. Integration of VEGF and α-SMA expression improves the prediction accuracy of fibrosis in chronic hepatitis C liver biopsy. Appl Immunohistochem Mol Morphol. 2017;25(4):261–70. 26990742 10.1097/PAI.0000000000000299

[30] Chan YH. Biostatistics 102: quantitative data – parametric & non-parametric tests. Singapore Med J. 2003;44(8):391–6. 14700417

[31] Chan YH. Biostatistics 103: qualitative data – tests of independence. Singapore Med J. 2003;44(10):498–503. 15024452

[32] Chan YH. Biostatistics 104: correlational analysis. Singapore Med J. 2003;44(12):614–9. 14770254

[33] Dimitriadis GD, Maratou E, Kountouri A, Board M, Lambadiari V. Regulation of postabsorptive and postprandial glucose metabolism by insulin-dependent and insulin-independent mechanisms: an integrative approach. Nutrients. 2021;13(1):159. 33419065 10.3390/nu13010159 PMC7825450

[34] Soon GS, Torbenson M. The liver and glycogen: in sickness and in health. Int J Mol Sci. 2023;24(7):6133. 37047105 10.3390/ijms24076133 PMC10094386

[35] Petersen MC, Vatner DF, Shulman GI. Regulation of hepatic glucose metabolism in health and disease. Nat Rev Endocrinol. 2017;13(10):572–87. 28731034 10.1038/nrendo.2017.80 PMC5777172

[36] Chemale G, Van Rossum AJ, Jefferies JR, Barrett J, Brophy PM, Ferreira HB. Proteomic analysis of the larval stage of the parasite, *Echinococcus granulosus*: causative agent of cystic hydatid disease. Proteomics. 2003;3(8):1633–6. 12923787 10.1002/pmic.200300487

[37] Hemer S, Konrad C, Spiliotis M, Koziol U, Schaack D, Förster S, et al. Host insulin stimulates *Echinococcus multilocularis* insulin signalling pathways and larval development. BMC Biol. 2014;12:1–22. 24468049 10.1186/1741-7007-12-5 PMC3923246

[38] Juyi L, Yan J, Xiufang W, Zhaoqing Z, Junliang L, Mingxing Z, et al. Analysis of the chemical components of hydatid fluid from *Echinococcus granulosus*. Rev Soc Bras Med Trop. 2013;46(5):605–10. 24270252 10.1590/0037-8682-0154-2013

[39] Pakala T, Molina M, Wu GY. Hepatic echinococcal cysts: a review. J Clin Transl Hepatol. 2016;4(1):39–46. 27047771 10.14218/JCTH.2015.00036 PMC4807142

[40] Loos JA, Negro PS, Ortega HH, Salinas FJ, Arán M, Pellizza L, et al. Anti-echinococcal effect of metformin in advanced experimental cystic echinococcosis: reprogrammed intermediary carbon metabolism in the parasite. Antimicrob Agents Chemother. 2024;68(10):e00941-24. 39264188 10.1128/aac.00941-24 PMC11459915

[41] Liu GS, Peng XY, Wu XW, Zhao J, Zhang YG, Zhang SJ, et al. Expression of α-SMA in the surrounding tissues of hepatic hydatid cyst. 2012.

[42] Kamm DR, McCommis KS. Hepatic stellate cells in physiology and pathology. J Physiol. 2022;600(8):1825–37. 35307840 10.1113/JP281061 PMC9012702

[43] Xie Z, Li Y, Xiao P, Ke S. GATA3 promotes the autophagy and activation of hepatic stellate cell in hepatic fibrosis via regulating miR-370/HMGB1 pathway. Gastroenterol Hepatol. 2024;47(3):219–29. 37207965 10.1016/j.gastrohep.2023.05.005

[44] Ahmad Z, Uddin N, Memon W, Abdul-Ghafar J, Ahmed A. Intrahepatic biliary cystadenoma mimicking hydatid cyst of liver: a clinicopathologic study of six cases. J Med Case Rep. 2017;11(1):317. 10.1186/s13256-017-1481-2. 29121977 10.1186/s13256-017-1481-2 PMC5680786

[45] Sufleţel RT, Stanca Melincovici C, Orăşan OH, Zaharie T, Gheban BA, Istrate A, et al. Activated hepatic stellate cells (Ito cells)-marker of advanced fibrosis in chronic viral hepatitis C: a pilot study. *J Gastrointestin Liver Dis*. 2023;32(2). 34 10.15403/jgld-4726 37345607

[46] Zhu J, Zhou T, Menggen M, Aimulajiang K, Wen H. Ghrelin regulating liver activity and its potential effects on liver fibrosis and echinococcosis. Front Cell Infect Microbiol. 2024;13:1324134. 38259969 10.3389/fcimb.2023.1324134 PMC10800934

[47] Shousha S, Gadir F, Peston D, Bansi D, Thillainaygam AV, Murray-Lyon IM. CD10 immunostaining of bile canaliculi in liver biopsies: change of staining pattern with the development of cirrhosis. Histopathology. 2004;45(4):335–42. 15469471 10.1111/j.1365-2559.2004.01927.x

[48] Kawabata H, Suzuki H, Takei J, Kaneko MK, Kato Y. Epitope mapping of an anti-CD10 monoclonal antibody (MME/1870) using enzyme-linked immunosorbent assay. Monoclon Antib Immunodiagn Immunother. 2022;41(1):15–9. 35225664 10.1089/mab.2021.0046

[49] Huang X, He C, Lin G, Lu L, Xing K, Hua X, et al. Induced CD10 expression during monocyte-to-macrophage differentiation identifies a unique subset of macrophages in pancreatic ductal adenocarcinoma. Biochem Biophys Res Commun. 2020;524(4):1064–71. 32070494 10.1016/j.bbrc.2020.02.042

[50] Brandau S, Hartl D. Lost in neutrophil heterogeneity? CD10! Blood. 2017;129(10):1240–1. 28280045 10.1182/blood-2017-01-761585

[51] Huapaya JA, Higgins J, Kanth S, Demirkale CY, Gairhe S, Aboye EA, et al. Vaccination ameliorates cellular inflammatory responses in SARS-CoV-2 breakthrough infections. J Infect Dis. 2023;228(1):46–58. 36801946 10.1093/infdis/jiad045 PMC10304754

[52] Xie L, Takahara M, Nakahara T, Oba J, Uchi H, Takeuchi S, et al. CD10-bearing fibroblasts may inhibit skin inflammation by down-modulating substance P. Arch Dermatol Res. 2011;303(1):49–55. 21076839 10.1007/s00403-010-1093-9

[53] Takele Y, Adem E, Mulaw T, Müller I, Cotton JA, Kropf P. Following successful anti-leishmanial treatment, neutrophil counts, CD10 expression and phagocytic capacity remain reduced in visceral leishmaniasis patients co-infected with HIV. PLoS Negl Trop Dis. 2022;16(8):e0010681. 35969625 10.1371/journal.pntd.0010681 PMC9410551

[54] Miyagawa Y, Fujiwara-Tani R, Ikemoto A, Sasaki R, Ogata R, Nishiguchi Y, et al. Significance of CD10 for mucosal immunomodulation by β-casomorphin-7 in exacerbation of ulcerative colitis. Curr Issues Mol Biol. 2024;46(7):6472–88. 39057028 10.3390/cimb46070386 PMC11276523

[55] Mishra D, Singh S, Narayan G. Role of B cell development marker CD10 in cancer progression and prognosis. Mol Biol Int. 2016;2016:4328697. 27965895 10.1155/2016/4328697 PMC5124668

[56] Wang S, Xiao Y, An X, Luo L, Gong K, Yu D. A comprehensive review of the literature on CD10: its function, clinical application, and prospects. Front Pharmacol. 2024;15:1336310. 38389922 10.3389/fphar.2024.1336310 PMC10881666

